# Implementation of respiratory‐gated VMAT on a Versa HD linear accelerator

**DOI:** 10.1002/acm2.12160

**Published:** 2017-08-18

**Authors:** Jeffrey E. Snyder, Ryan T. Flynn, Daniel E. Hyer

**Affiliations:** ^1^ Department of Radiation Oncology University of Iowa Hospitals and Clinics Iowa City IA 52242 USA

**Keywords:** Anzai, Elekta, gating, response interface, VMAT

## Abstract

The accurate delivery of respiratory‐gated volumetric modulated arc therapy (VMAT) treatment plans presents a challenge since the gantry rotation and collimator leaves must be repeatedly stopped and set into motion during each breathing cycle. In this study, we present the commissioning process for an Anzai gating system (AZ‐733VI) on an Elekta Versa HD linear accelerator and make recommendations for successful clinical implementation. The commissioning tests include central axis dose consistency, profile consistency, gating beam‐on/off delay, and comparison of gated versus nongated gamma pass rates for patient‐specific quality assurance using four clinically commissioned photon energies: 6 MV, 6 FFF, 10 MV, and 10 FFF. The central axis dose constancy between gated and nongated deliveries was within 0.6% for all energies and the analysis of open field profiles for gated and nongated deliveries showed an agreement of 97.8% or greater when evaluated with a percent difference criteria of 1%. The measurement of the beam‐on/off delay was done by evaluating images of a moving ball‐bearing phantom triggered by the gating system and average beam‐on delays of 0.22–0.29 s were observed. No measurable beam‐off delay was present. Measurements of gated VMAT dose distributions resulted in decrements as high as 9% in the gamma passing rate as compared to nongated deliveries when evaluated against the planned dose distribution at 3%/3 mm. By decreasing the dose rate, which decreases the gantry speed during gated delivery, the gamma passing rates of gated and nongated treatments can be made equivalent. We present an empirically derived formula to limit the maximum dose rate during VMAT deliveries and show that by implementing a reduced dose rate, a gamma passing rate of greater than 95% (3%/3 mm) was obtained for all plan measurements.

## 1 INTRODUCTION

Respiratory gating in radiotherapy plays an important role in the treatment of moving targets as it allows the volume of normal tissue exposed to high doses of radiation to be reduced. This is made possible by energizing the beam only at preselected phases of the breathing cycle, reducing the amount of tumor motion that needs to be accounted for in the treatment plan and shrinking the size of the internal target volume (ITV).^1^ The benefits of a smaller ITV and additional normal tissue sparing are dosimetrically appealing, especially for targets in the pancreas, liver, and lung which can exhibit 2–3 cm of respiratory motion.^2^, ^3^ For these sites, gated radiotherapy may reduce pulmonary, cardiac, and esophageal toxicity compared to nongated radiotherapy.^4^, ^5^

The most commonly used respiratory gating systems rely on external markers to generate the respiratory trace needed for gating the treatment beam. One such system, the AZ‐733VI by Anzai Medical Systems (Tokyo, Japan), uses a load cell and elastic belt wrapped around the patient's abdomen to monitor respiration.^6^ During inhalation, the elastic belt stretches with expansion of the patient's abdomen and the compressive forces are registered on the load cell. The signal from the load cell is correlated to tumor motion through a retrospective 4‐D computed tomography (CT) scan at the time of simulation.

The combination of respiratory gating and volumetric modulated arc therapy (VMAT) presents additional complexities to the radiation delivery process as the dose rate and gantry speed must be repeatedly ramped up and halted during each breathing cycle. The repeated ramp up presents challenges for flattening filter free (FFF) modes of delivery in which high dose rates, and often fast gantry rotation speeds, are used. While gated VMAT delivery is of interest in the literature,^7^, ^8^ there is limited work on clinical implementation of this technology.^9^, ^10^ Specifically, no literature exists examining the delivery accuracy of gated VMAT FFF treatments, particularly at different dose and fractions with varying gating windows.

This work focuses on the clinical implementation of gated VMAT deliveries on an Elekta Versa HD accelerator with the Anzai AZ‐733VI system. Both flattened and FFF delivery modes were investigated, and limitations as well as recommendations are presented.

## 2 METHODS

### 2.A Beam tuning parameters

Based on recommendations by the manufacturer and those found in the literature, several linac parameters were modified to achieve optimal gating performance. The gun filament current hold‐on delay was set to 6.5 s. This parameter represents the amount of time that the electron gun will remain at its operating current following a beam‐on interruption. Failure to adjust this parameter would lead to significantly longer beam‐on delay times, as described by Cui et al.^9^ The gun filament current off‐frequency delay, which is set to 0 s by default, was increased to 1.5 s. This parameter keeps the gun at its operating current for an extended time if the magnetron frequency is out of tolerance. The magnetron tuner control item for gun delay was also adjusted and decreased to 0 s from a default of 1 s, allowing the electron gun to turn on as soon as the magnetron frequency is in tolerance rather than waiting through a delay. Lastly, the magnetron tuner servo gain was adjusted to ensure that the dose rate ramps up smoothly when the beam turns on. This is an important parameter because there are numerous beam stops during gated deliveries and any inconsistencies in the dose rate ramp up could significantly impact the motion of the gantry and multileaf collimator (MLC).

### 2.B Respiratory waveform

The Anzai patient belt and load cell were secured to a Quasar respiratory phantom (Modus Medical Devices Inc., London, ON, Canada), and respiratory waveforms of varying frequency were recorded for use throughout this study. All respiratory waveforms followed a cos^6^ function to represent a nominal breathing pattern.^9^

### 2.C Central axis dose output

**Figure 1:**
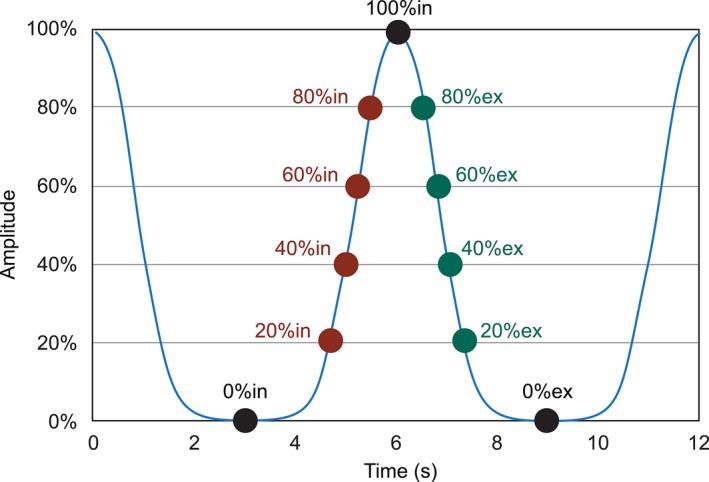
Nomenclature used to define phases in a respiratory breathing trace.

Central axis dose output was evaluated for both gated and nongated treatment deliveries with breathing rates of 10 breaths per minute (bpm) and 15 bpm and gating windows of 20% exhale (ex) to 20% inhale (in) and 80%ex to 80%in, covering the range of respiratory rates and gating windows commonly seen in our clinic. Figure 1 shows a respiratory waveform depicting the nomenclature used for defining gating windows throughout this study. The central axis dose output was measured using a 0.125 cc ion chamber in a 30 × 30 cm^2^ solid water phantom with a 100 cm source to surface distance (SSD), 10 cm depth, and 10 × 10 cm^2^ field‐size. All treatments were delivered using an Elekta Versa HD and measurements were made for 6 MV, 6 FFF, 10 MV, and 10 FFF.

### 2.D Profile consistency

Consistency of the radiation profile between gated and nongated deliveries was evaluated during an arc delivery. The Sun Nuclear MapCheck2 (Sun Nuclear Corporation, Melbourne, FL, USA) was fixed to the gantry via a gantry mounting fixture specifically designed for use with the Mapcheck2 in order to maintain an *en face* orientation during rotational exposure. Beams with energies of 6 MV, 6 FFF, 10 MV, and 10 FFF were delivered in a single 360° arc with a 20 × 20 cm^2^ field size and solid water was added such that the profiles were measured at a depth of 3 cm. The maximum nominal dose rate for each beam energy was used and monitor units were chosen such that the gantry would rotate at a maximum speed of 6° per second. A breathing rate of 15 bpm and a 20%ex–20%in gating window was used for all energies. Agreement between gated and nongated deliveries was evaluated with a percent difference criterion of 1% for doses above a threshold of 50% using global normalization.

### 2.E Time delay

The time delay during gated delivery is defined as the time between when the beam‐on signal is sent to the linac until the radiation beam actually turns on and likewise the time between when the beam‐off signal is sent until the time in which the beam turns off. This is important to measure during the commissioning process because this parameter can have a dosimetric impact on delivered plans, especially if a beam‐off delay is found.

**Figure 2:**
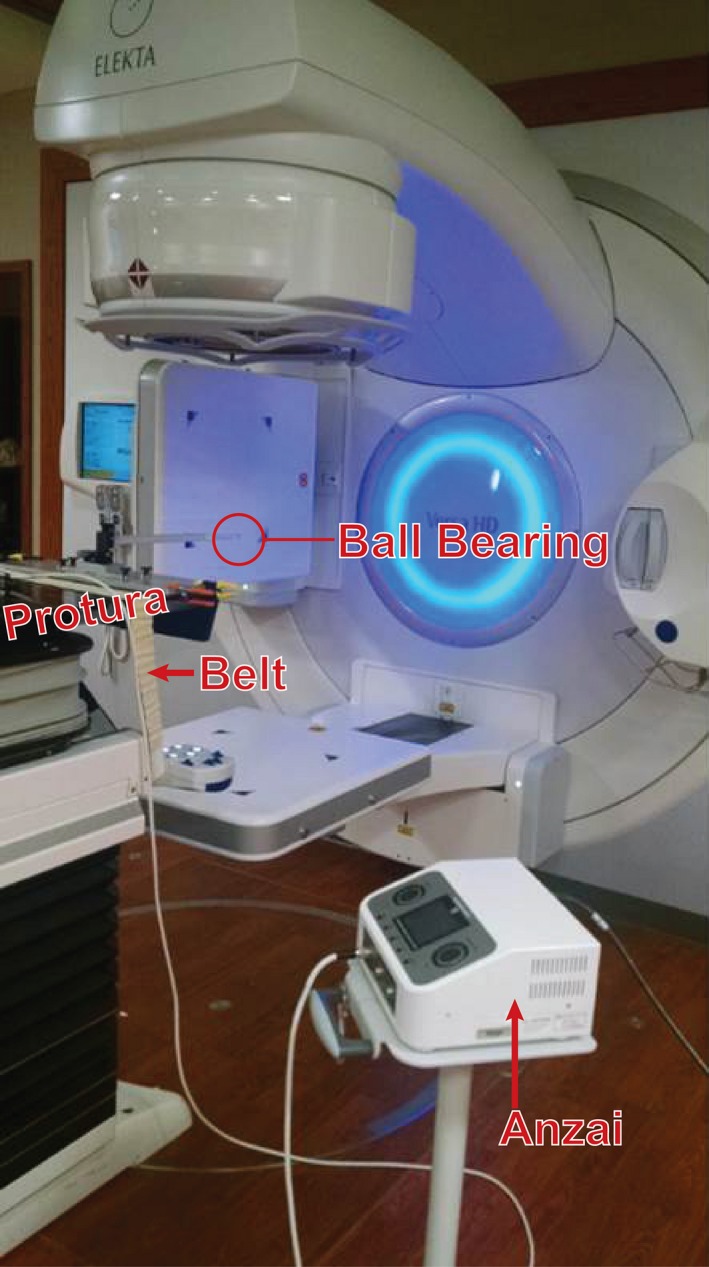
Setup used to measure beam on and off delay time during gated deliveries.

To measure the beam‐on and beam‐off delay time, a Protura six degree of freedom robotic couch (CIVCO Medical Solutions, Kalona, IA, USA) was programmed to move in a sinusoidal pattern with an amplitude of 20 mm in the lateral direction and with a frequency of 15 cycles per minute. An Anzai belt with a load cell was secured to the couch and a ball bearing phantom was placed at isocenter. With this configuration, the Anzai system could be used to record a respiratory trace based on the motion of the couch and to trigger the beam at a selected respiratory phase. By keeping the electronic portal imaging device (EPID) in a ready state, images of the ball bearing phantom were captured anytime the beam was energized. This setup is shown in Fig. 2. To begin the experiment, the couch was moved to various predefined positions and stationary images of the ball bearing phantom were captured. These stationary images provided comparison points for images acquired with the moving ball bearing phantom. The stationary position acquired images were also used to create a relationship between the physical position of the ball bearing phantom and the amplitude in the breathing trace to which that position corresponded. This position versus amplitude relationship, combined with amplitude versus time data that was exported from the recorded breathing trace, allowed a correlation between position of the ball bearing phantom and time to be developed. For implementation in a broader range of clinics, this test could also be performed using a Quasar phantom or a motion phantom, sold by Anzai, and attached to the patient belt and ball bearing phantom then onto the motion platform to trigger the gating system in a similar manner.

The ImageJ software package was used to analyze the acquired MV EPID images. Differences in position between a stationary image and an image acquired from triggering the gating system for the same amplitude were compared. These differences in distance were then used to calculate beam‐on and beam‐off time delays. Continuously acquired EPID images of the moving ball bearing phantom were compared to stationary images taken at 0%ex and 100%in, and this analysis served as a control. Two different gating windows of 10%in–90%in and 25%in–75%in were measured. These gating windows were chosen because they represent high velocity regions within the breathing cycle which helps make the beam‐on and off delays more apparent. For each gating window, four different trials were performed, and the average beam‐on delay and standard deviation is reported.

### 2.F Gated patient plan verification

The plan quality between gated and nongated deliveries was evaluated using a Sun Nuclear ArcCheck (Sun Nuclear Corporation, Melbourne, FL, USA). A single celiac VMAT plan (patient 1), which consisted of one 360° arc and a PTV volume of 51.97 cm^3^, was recomputed for each energy of interest (6 MV, 10 MV, 6 FFF, and 10 FFF) as an initial evaluation. Each plan used the same control points and only the monitor units were scaled such that an equivalent dose would be delivered for each energy of interest. Both gated and nongated measurements were compared to plans calculated using the Pinnacle treatment planning system (TPS). Pass rates were calculated using an absolute gamma analysis with passing criteria of 3% and 3 mm with a dose threshold of 10%. Sun Nuclear's commercially available SNC Patient software was used for this analysis and the percentage of passing points was compared for gated and nongated treatment deliveries. The ArcCheck also allows the insertion of a 0.125 cc ion chamber, which can be used to obtain a point dose measurement within the plan. The point of measurement was chosen to be within a low dose gradient (less than 3% over the dimension of the chamber) region of the plan. The same point was chosen for gated and nongated deliveries and the results were compared.

**Figure 3:**
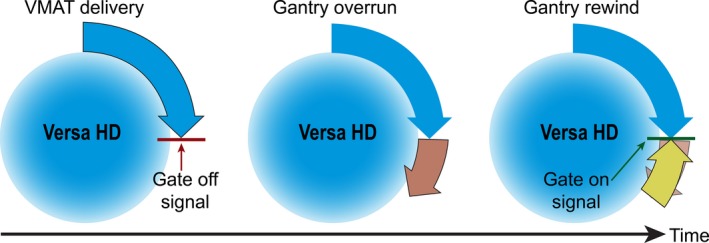
Schematic of gantry overrun after receiving gate‐off signal and subsequent reversal of direction before the next gate‐on signal is received.

During gated deliveries, it was noted that the gantry's momentum resulted in continued rotation after the gate‐off signal has been received and the radiation beam had turned off. Due to this continued rotation, the gantry must reverse direction such that it is in position before the next gate‐on signal is received and the radiation beam is resumed. Figure 3 shows a schematic of this phenomenon. Due to this observation, the effects of the gating window length and gantry speed during VMAT delivery were also investigated. The effect of gating window length was evaluated using patient 1 and a second patient (patient 2) with an abdominal tumor with a PTV volume of 17.66 cm^3^. To investigate the effects of gantry speed, several methods were used which include increasing the dose per fraction of the plan, physically reducing the maximum gantry speed through a parameter in service mode, and by reducing the maximum dose rate. Either increasing the dose per fraction or decreasing the dose rate has the same secondary effect of decreasing the gantry speed.

To further validate the effect of dose rate on gated VMAT plan deliveries, four plans from previously treated patients with tumors in the abdominal region were delivered with gating at the nominal dose rate, nongated at the nominal dose rate, and gated at a reduced dose rate. The nominal dose rates are 600 MU/min, 500 MU/min, 1500 MU/min, and 2300 MU/min for 6 MV, 10 MV, 6 FFF, and 10 FFF, respectively. The gamma pass rate of each of these plans was compared for all three cases. The PTV volumes for these plans were 227.09 cm^3^, 214.37 cm^3^, 262.22 cm^3^, and 27.2 cm^3^ for 6 MV, 10 MV, 6 FFF, and 10 FFF, respectively. Each of these patients had received four‐dimensional CT scans but were not gated during their initial treatment due to limited tumor motion. These sites, however, were representative of treatment regions which are commonly gated. All VMAT patient plans measured in this study were at 12 bpm unless otherwise noted.

### 2.G Clinical implementation

After the completion of the initial commissioning process, the gamma passing rates of clinical patients who received gated VMAT treatments continued to be monitored. An absolute gamma analysis with passing criteria of 3% and 3 mm with a 10% dose threshold was used. Patient pretreatment quality assurance was completed under gated machine operation using a prerecorded breathing trace of 12 bpm. Gating windows were chosen to match the clinically indicated gating window for each patient. The range of gating windows used in these measurements was from 40%ex–30%in to 60%ex–60%in. All treatment plans were measured using a reduced dose rate. Six patient plans have been measured with two plans measured at 6 MV energy, two at 10 MV energy, and two at 10 FFF energy.

## 3 RESULTS

### 3.A Central axis dose output

**Figure 4:**
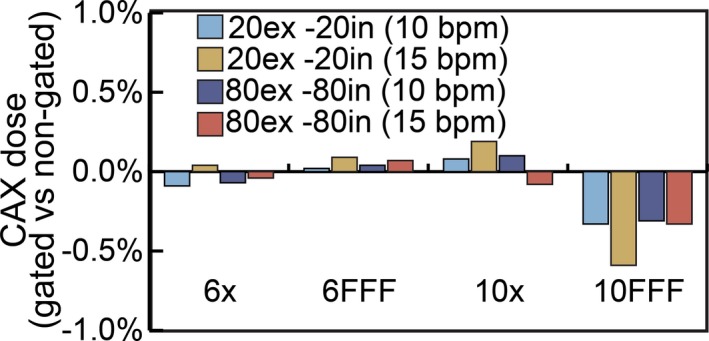
Comparison of central axis dose output for gated and nongated treatment deliveries.

The dose output along the central beam axis was measured during gated and nongated deliveries for 6 MV, 6 FFF, 10 MV, and 10 FF energies. Figure 4 shows these results for 10 and 15 bpm and gating windows of 20%ex–20%in and 80%ex–80%in. The results presented are the percent difference from the nongated delivery. The largest percent difference from the nongated delivery is −0.59% for 10 FFF energy with a gating window of 20%ex–20%in and 15 bpm. For the 80%ex–80%in gating window, the largest difference was −0.33% which occurred using the 10 FFF energy and 15 bpm. The maximum difference among each energy is −0.09%, 0.09%, 0.19%, and −0.59% for 6 MV, 6 FFF, 10 MV, and 10 FFF energies, respectively.

### 3.B Profile consistency

**Figure 5:**
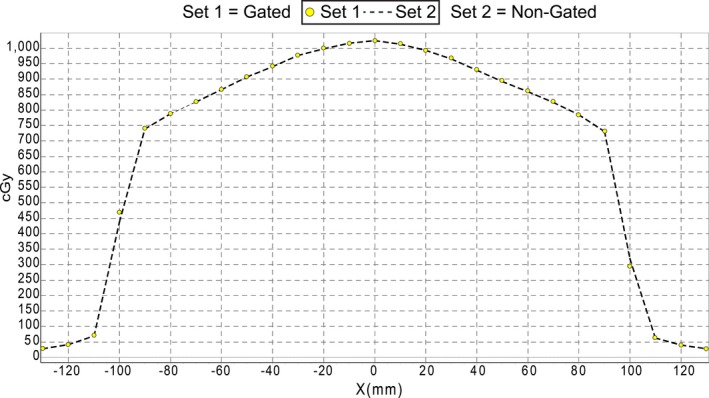
Analysis of profile consistency for 6 FFF energy. Agreement between gated and nongated deliveries was evaluated with a percent difference criteria of 1% and a dose threshold of 50%. The yellow circles represent points on the gated profile and the dashed line is measured nongated data.

Figure 5 shows a comparison between gated and nongated deliveries of a 20 × 20 cm^2^ open field profile for the 6 FFF energy. The minimum pass rate for all energies was 97.8% which occurred at 6 MV when comparing gated and nongated deliveries with a percent difference criteria of 1% and a dose threshold of 50%. All other energies passed with 100% agreement.

### 3.C Time delay

**Figure 6:**
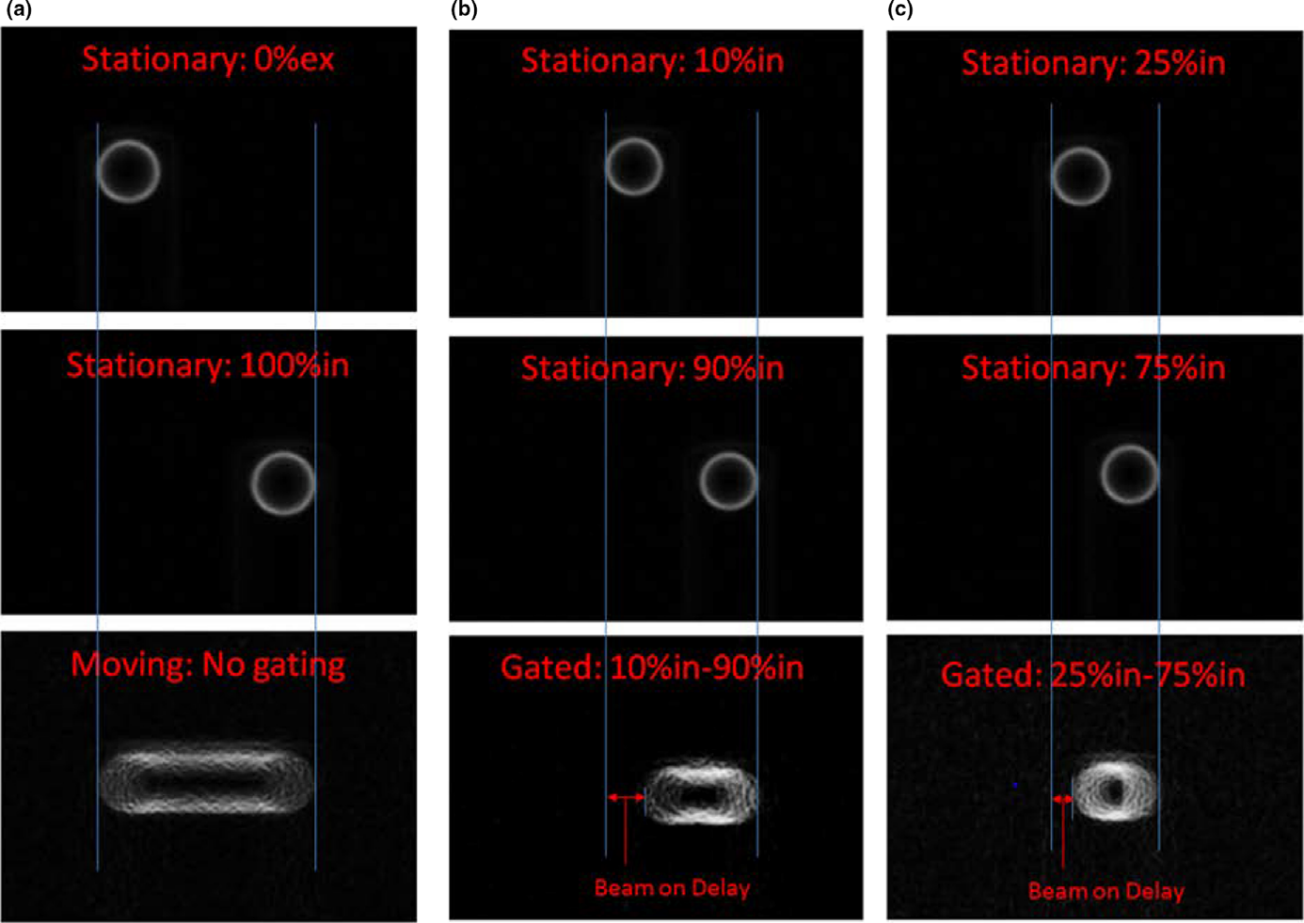
Gating delay time. (a) Stationary images acquired at 0%ex and 100%in and a continuous image acquired of the moving phantom with no gating. (b) Stationary images acquired at 10%in and 90%in and a continuous image of the moving phantom acquired during the gating window of 10%in‐90%in in which the beam‐on delay can be visualized. (c) Shows the same analysis as in (b) but for the gating window of 25%in–75%in.

EPID images of the moving ball bearing phantom were acquired for gating windows of 10%in–90%in and 25%in–75%in. The postprocessed EPID images, with delineations for expected and measured ball positions, are shown in Fig. 6. Based on these differences, the beam‐on delay was calculated. The control experiment with no gating (Fig. 6a) shows that the stationary EPID images and continuously acquired nongated images exhibit the same extent of motion. For the gating window of 10%in–90%in the beam‐on delay was measured to be 0.29 s ± 0.02 s and for 25%in–75%in the beam‐on delay was 0.22 ± 0.05 s. There was no observed beam‐off delay for either gating window.

### 3.D Gated patient plan verification

**Figure 7:**
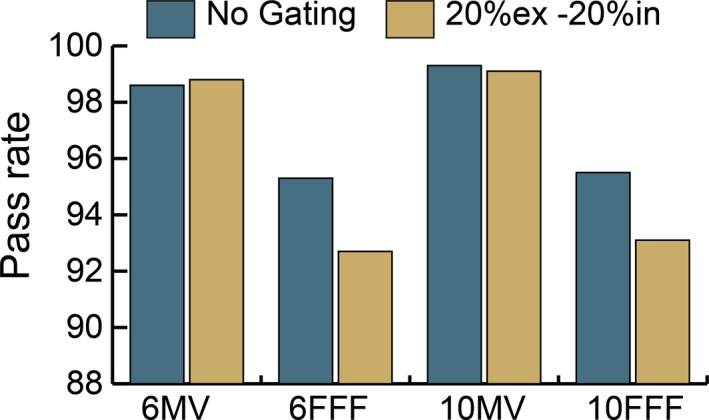
Gamma pass rate comparison between gated and nongated deliveries of the same plan at four clinical energies. Measured distributions evaluated against planned distribution at 3%/3 mm.

Gamma pass rates for the VMAT plan delivered for patient 1 at 6 Gy per fraction with a breathing rate of 15 bpm and nominal maximum dose rate with and without gating are shown in Fig. 7. Ion chamber readings collected within the ArcCheck phantom during the same deliveries were within 0.4% for all energies when comparing the gated and nongated results. The gated plan deliveries were approximately equivalent for the flattened beams but yielded a decrement in passing rate of up to 2.6% for the FFF beams.

**Figure 8:**
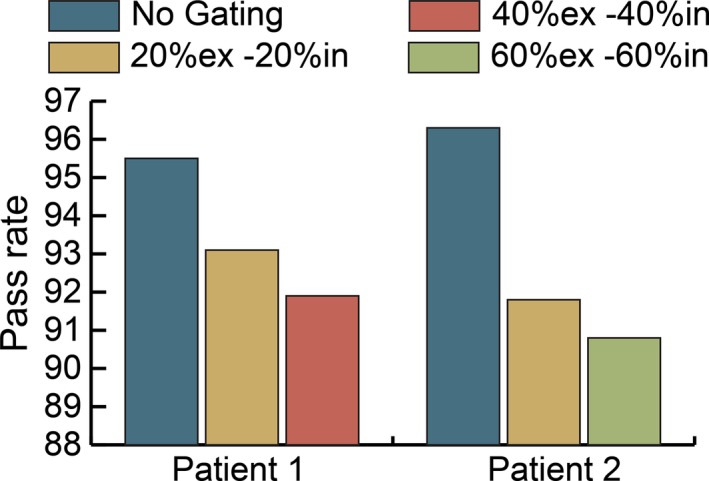
Effect of gating window on gamma pass rate at 10 FFF energy. Measured distributions evaluated against planned distribution at 3%/3 mm.

This test was repeated with varying gating windows using the 10 FFF energy for one additional patient (patient 2). The results are shown in Fig. 8. As the gating window was increased, the gamma passing rate decreased for both patients.

**Figure 9:**
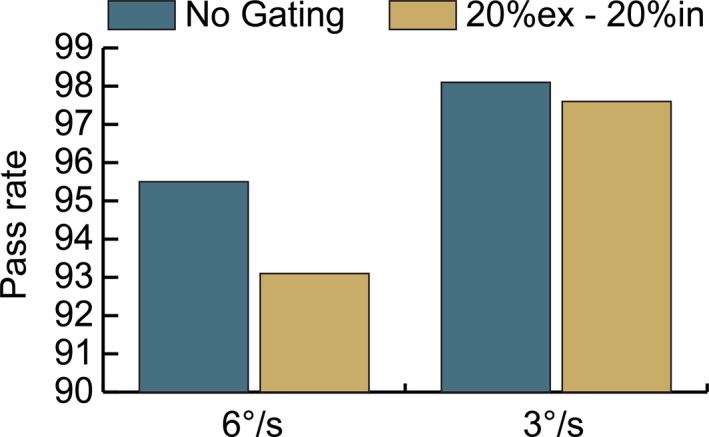
Effect of reduced gantry speed on gamma pass rate for patient 1 at 10 FFF energy. Measured distributions evaluated against planned distribution at 3%/3 mm.

Patient 1 was then re‐evaluated using the 10 FFF energy and reduced gantry speed. The maximum allowable gantry speed was changed from 6 degrees per second to 3 degrees per second. The results of this measurement are shown in Fig. 9. The passing rates of both the gated and nongated treatment deliveries increased at the slower gantry speed. At the faster gantry speed, the decrement from the gated treatment was 2.4% and the decrement from gating at the slower gantry speed was 0.5%.

**Figure 10:**
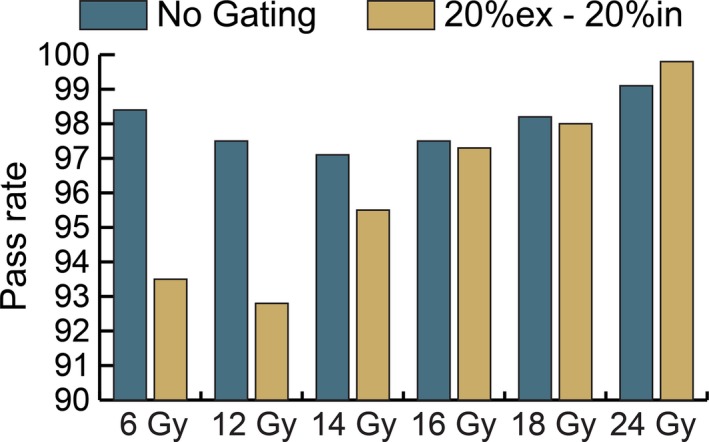
Effect of dose per fraction on gamma pass rate for patient 3 at 10 FFF energy. Measured distributions evaluated against planned distribution at 3%/3 mm.

A third patient was selected (patient 3) to investigate the total dose delivered per fraction versus pass rate. A 20%ex–20%in gating window was used and the maximum allowable gantry speed of six degrees per second was restored. The monitor units of the plan were scaled to achieve different doses but all control points within the plan remained constant. Doses of 6–24 Gy were tested using 10 FFF energy and the results are shown in Fig. 10.

**Figure 11:**
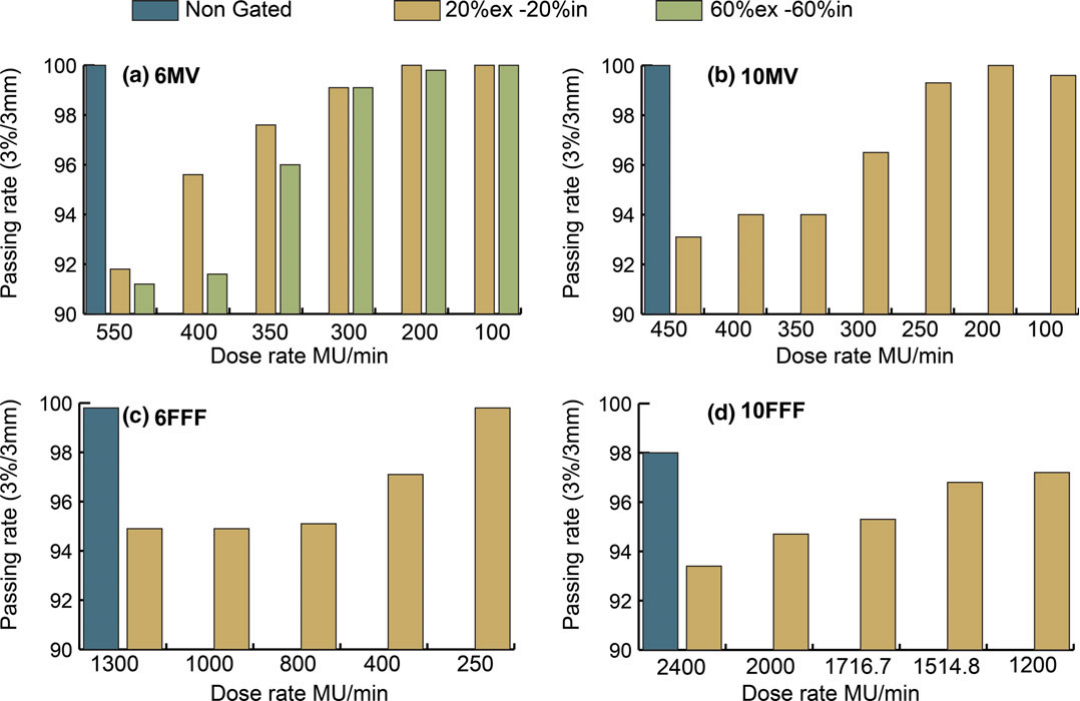
Maximum dose rate versus gamma passing rate. (a) 6 MV plan for patient 3 (2 Gy) with two gating windows (b) 10 MV plan for patient 3 (2 Gy) (c) 6 FFF patient plan (6 Gy), and (d) 10 FFF patient plan (7 Gy). Measured distributions evaluated against planned distribution at 3%/3 mm.

Another test performed to reduce the gantry speed without increasing the dose per fraction was to limit the maximum dose rate during delivery. The plan for patient 3, delivered at 2 Gy per fraction, was recomputed for 6 MV and 10 MV energies, and two additional plans were measured for 6 FFF and 10 FFF energies. The 6 FFF plan was delivered at 6 Gy per fraction and the 10 FFF plan was delivered at 7 Gy per fraction. The gamma pass rate was evaluated for each energy at various maximum dose rates and the results are shown in Fig. 11. The pass rate for the gated delivery increased as the dose rate decreased for each energy.

**Figure 12:**
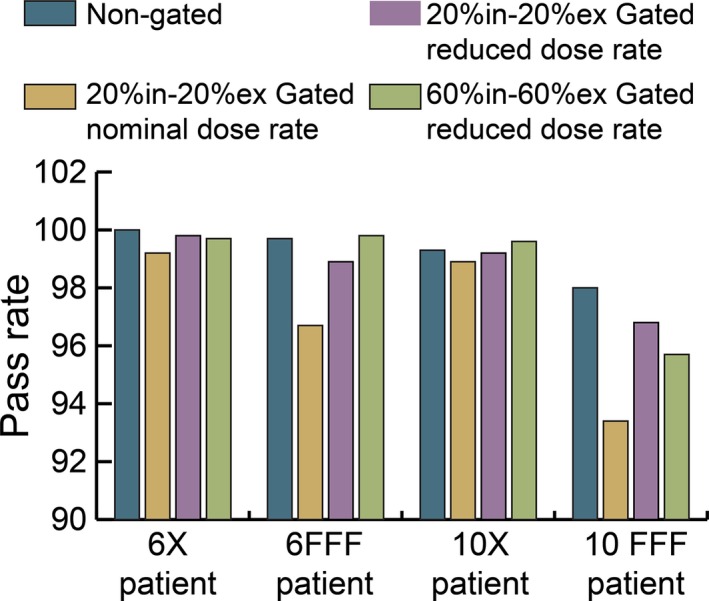
Gamma pass rates of four different patient plans at reduced maximum dose rate.

Four additional different patient plans (one at each energy level) were nongated, gated, and gated at reduced dose rate. The 6 MV and 10 MV plans were measured at 2 Gy per fraction and the 6 FFF and 10 FFF plans were measured at 6 Gy and 7 Gy per fraction, respectively. Reduced dose rates were calculated such that the time required to complete a 360° arc would be 1.7 min rather than the typical 1 min when running at full speed. These reduced dose rates were delivered with gating windows of 20%ex–20%in and 60%ex–60%in. These results are shown in Fig. 12. The reduced dose rate deliveries have a higher pass rate than the same treatments at nominal dose rates. All of the gated plans at reduced dose rate have a gamma pass rate of 95.7% or higher for all energies and gating windows measured.

**Figure 13:**
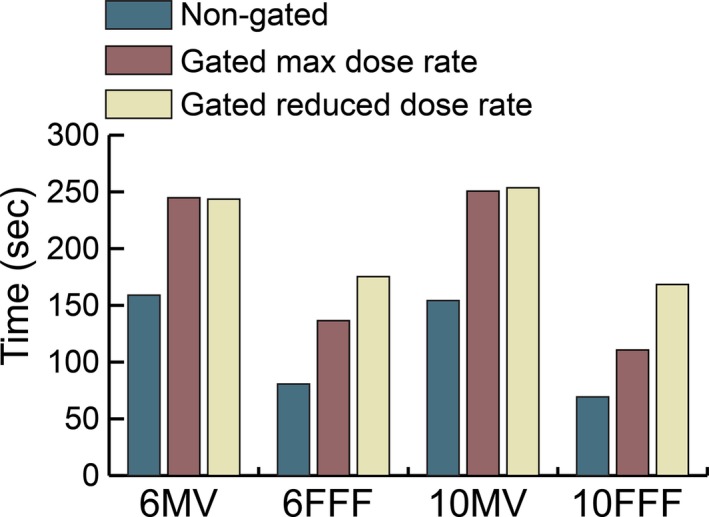
Impact on delivery time due to gating for patient 1.

The additional time required to deliver gated and nongated treatments was evaluated for each beam energy using patient 1, 6 Gy per fraction, and a 40%ex‐40%in gating window with a 12 bpm breathing rate. Gated treatments were delivered at nominal dose rate and a reduced dose rate. These results are shown in Fig. 13. The delivery time between gated nominal and gated with reduced dose rate treatment times were within one‐second for 6 MV and 10 MV. Time differences of 38.8 and 57.7 s were observed between nominal dose rate and reduced dose rate gated treatments for 6 FFF and 10 FFF, respectively. The maximum difference in delivery time for a nongated versus gated delivery was 99.4 s at 10 MV.

### 3.E Clinical implementation

To date, six clinical gated VMAT patient plans have been measured at our institution. The average gamma passing rate from these measurements is 98.6% with a standard deviation of 2.2%. The lowest passing rate was 94.2% which was observed for a 10 FFF patient with a gating window of 60%ex–40%in. All measurements were made using a reduced dose rate.

## 4 DISCUSSION

Respiratory gating plays an important role in radiotherapy, allowing additional sparing of normal tissue for moving targets by reducing the size of the target volume. For these benefits to be fully realized, the linear accelerator must be able to accurately deliver treatments when under gated operation. In this study, we evaluated several key components of commissioning an Anzai gating system for use with an Elekta Versa HD linear accelerator including central axis depth dose, dose profile consistency, beam‐on delay, and accuracy of delivered plans. Particular emphasis was placed on VMAT treatments with FFF beams and on developing methods to ensure accurate delivery for all cases.

The central axis dose output and beam profile consistency was evaluated for 6 MV, 6 FFF, 10 MV, and 10 FFF energies. The percent difference between gated and nongated deliveries was less than 0.6% among all gating windows and breathing rates tested. The beam profile consistency, (measured over a 360° arc for an open field), showed a 97.8% or better agreement between gated and nongated treatment deliveries. These results illustrate that for both stationary and simple arc fields the Elekta Versa HD is very stable under gated operation.

The average beam‐on and beam‐off delay during gated treatments is another important parameter to characterize during the commissioning of a gating system as these beam delays could have a dosimetric impact on plan delivery. The average beam‐on delay was 0.29 and 0.22 s for 10%in–90%in and 25%in–75%in gating windows, respectively. These results are consistent with Cui et al.^9^ who reported a beam‐on delay of 0.22 s or less. There was no measurable beam‐off delay for either gating window. These data show that the target would never leave the ITV and that less motion is being treated than accounted for during planning. This is a conservative approach as any beam‐off delays would have a more severe dosimetric impact because the target would leave the ITV and more normal tissue than expected would be treated.

While gated and nongated treatment deliveries may have good agreement for open and stationary fields, VMAT treatments provide unique challenges which include varying gantry speeds and dose rates, and thus plan comparisons must be done during the commissioning process of a new gating system. Figure 7 shows the decrement in the gamma passing rate of plans measured with and without gating. The pass rate decrease is more substantial for FFF treatments in which the gantry more frequently rotates at maximum speed because of the high dose rates available. The problem with a high gantry speed is that the momentum causes the gantry to overrun its intended stop position when the gate‐off signal is received and the gantry must reverse direction to get back into position before the next gate‐on signal is received. It was observed that at times the momentum of the gantry was carrying it in the opposite direction than intended in the treatment plan when the next gate‐on signal was sent, forcing the gantry to abruptly change direction before moving in the intended plan direction. We hypothesized that these sudden changes of direction led to the decrement in passing rates of gated treatments. Our hypothesis is true based on the results shown in Fig. 8 in which the gating window of the plan was increased and resulted in decreased gamma pass rates. Larger gating windows provide less time for the gantry to rewind and return to its planned position prior to the next gate‐on signal being sent.

To further investigate the effect of gantry speed on the passing rate of gated treatments, the speed of the gantry was reduced in several ways including reducing the maximum allowable gantry speed through a setting in service mode, increasing the dose per fraction, and reducing the maximum dose rate of the plan. For each method, the gated gamma pass rates increased for deliveries in which the speed of the gantry was reduced. The results in Fig. 10 show that the gamma pass rate increased as the dose per fraction was increased. If the dose per fraction is large enough, the gantry will not rotate at full speed even when the full dose rate is utilized. The results in Fig. 11 show that the gamma pass rate can also be increased by setting a maximum limit on the dose rate used during delivery and this approach can be effective even at a small dose per fraction, such as 2 Gy.

Of the methods used to reduce the gantry speed, we have chosen to reduce the maximum dose rate of VMAT plans for clinical implementation. If maximum dose rate and gantry speed is maintained, significant detriment in the passing rate of gated plans would be present for all but highly hypofractionated dose regiments, especially when using the FFF beams. This would greatly limit the applicability of gating. Limiting the maximum gantry speed, while effective, would require a reduced gantry speed for all patients treated including those who do not require gating. A reduction in gantry speed cannot be selectively applied to only gated patients in the Elekta control software. Therefore, the method of reducing dose rate was chosen for clinical implementation because of its effectiveness in increasing gamma pass rates and because it can be selectively applied to only gated patients. We have empirically derived a formula, shown in Eq. (1), for the maximum allowable dose rate for VMAT treatments by analyzing the data shown in Fig. 11.

(1) $$MU/min\leq \frac{Arc\;MU}{Arc\;Angle}\times \frac{360^{\circ }}{1.7\;min}$$

This equation is equivalent to a gantry speed of approximately 3.5° per second. By testing each energy with variable maximum dose rates, as described in Fig. 11, we were able to determine an acceptable upper threshold for dose rate which corresponds to a maximum gantry speed. The equation was developed such that the maximum dose rate used would yield a pass rate of 95% or greater for each energy. This was further tested and validated as shown in Fig. 12 where an additional patient at each energy was measured nongated, gated at nominal dose rate, and gated with a reduced dose rate empirically derived using Eq. (1). All of the measured plans passed at greater than 95% for gating windows of 20%ex–20%in and 60%ex–60%in. Since the clinical release of gated VMAT patient deliveries at our institution, all but one plan measured using the reduced dose rate shown in Eq. (1) has had a gamma passing rate of 95% or greater. A single plan using 10 FFF energy and a gating window of 60%ex–40%in had a gamma passing rate of 94.2% which was deemed clinically acceptable.

The increased delivery time due to gating and gating with reduced dose rate is shown in Fig. 13. These plans were delivered at 6 Gy and for energies of 6 MV and 10 MV the delivery time for gated treatments at nominal and reduced rate is within 1 s of each other. This result illustrates that the maximum allowed dose rate will only limit the delivered dose rate in certain circumstances and certain periods within the delivery. For some hypofractionated plans, the calculated maximum allowable dose rate from Eq. (1) will be greater than the maximum available dose rate at that energy, thus having no impact on delivery time. Furthermore, the time limitation from reduced dose rate will be highly plan dependent as some plans never utilize the maximum dose rate and few plans use maximum dose rate over their entire planned arc length.

## 5 CONCLUSION

On Elekta Versa HD linear accelerators, high gantry velocity can yield a significant decrement in the gamma passing rate of gated VMAT plans compared to that of nongated deliveries. This difficulty can be overcome by imposing a maximum dose rate for gated treatments. All plans tested at the recommended maximum dose rate pass at greater than 95% for all gating windows evaluated.

## CONFLICT OF INTEREST

All authors have declared that they have no pertinent conflicts of interest with this work.
